# Editorial for the Special Issue on Micro-Manufacturing and Applications

**DOI:** 10.3390/mi12080851

**Published:** 2021-07-21

**Authors:** Atanas Ivanov

**Affiliations:** Department MAE, College CDEPS, Brunel University, London UB8 3PH, UK; Atanas.Ivanov@brunel.ac.uk

This editorial is for collating the Special Issue on micro-manufacturing and applications, based on stringently selected papers presented to the editorial board of this Special Issue. All papers selected have undertaken a thorough peer-review process through a rigorous review system. There are 11 papers finally included in this issue.

Micro-manufacturing processes have been known for quite some time now, but their applications were often initially limited in the hands of researchers and developers. With the constantly growing demand for micro and nano systems on the market, these technologies are increasingly finding their natural fields of applications. Their rapid growth, not only as R&D tools but also as real industry applicable technologies, means that the process became irreversible and these technologies have earned their place, not only in the labs but at real manufacturing set-ups.

Micro- and nano-manufacturing has become a key value-added engineering, enabling technology for modern, advanced manufacturing. More and more micro- and nano-technologies reach their maturity and become part of the industrially applied technologies in batch and mass production of micro and nano products. Micro- and nano-manufacturing technologies are now widely used in many sectors of the engineering industry such as automotive, electronics, medical, and many other sectors. New technologies have captured the interest, not only of researchers but also high-tech industries. The commercial advantages of employing these new emerging manufacturing technologies are not always clear in the beginning but allows companies to secure specific micro/nano niches in the marketplace and later to work on technological improvements and broadening the application field.

The main products were related to problems in making and exploiting micro-channels, micro-optics, MEMS, and material properties for micro structuring applications. The suggested new technology for micro-wire electrochemical discharge machining (WECDM), which is proposed as ultrasonic vibration assisted WECDM with a micro helical electrode [1], addresses the need for the production of high aspect ratio features for MEMS structures. The focus of this Special Issue, again, the development and challenges associated with emerging new micro features inspired by nature, and finding ways they can be manufactured in a sustainable and economic way [2]. Nowadays, with the rapid development of the electronic industry, it is becoming increasingly important that the removal of heath from electronic components is fast and effective. For that purpose, micro channels in porous media were investigated [3], concluding with a more effective, two-phase heath exchanging opportunity. The wire EDM process was employed for machining thin fins [4], which can be used for many purposes, one being to serve as heath exchanges and/or micro-fluidic systems. This investigation aims to determine the process capabilities and fields of application of the proposed method.

Multifocal micro-lens arrays technology is also presented with the aim to target 3D imaging, depth sensors and development, and wide field of view cameras [5]. The paper reports the fabrication method of high NA, Mf-MLAs for the extended depth-of-field, using single-step photolithography assisted by chemical wet etching.

Material structuring plays a crucial role in surface topography and, hence, the appearance and possibility of introducing micro features or micro elements in these surfaces [6]. Here, the surface topography transfer mechanism and microconvex change law during cold rolling are revealed with the purpose to improve the surface topography.

The process of etching for the production of micro and nano features or particles is, again, a very important part of this paper selection, demonstrating the use of Integrated Diffused Silicon Two-Zone Heaters for Silicon-Pyrex Glass Microreactors for Production of Nanoparticles [7]. The obtained results show that the proposed procedure for the heater fabrication is robust, stable, and controllable, with a decreased sensitivity to random variations of fabrication process parameters. Compared to metallic or polysilicon heaters typically integrated into microreactors, this approach offers improved control over heater characteristics through the adjustment of the Boron doping level and profile.

Nowadays, life seems impossible without our smart phones and all other gadgets of commodity. The majority of them contain elements and systems produced in many millions and fault testing is compulsory before assembly to minimise the lost function effect. A typical representative in this group is the miniature vibration motors and vibrating mechanisms in our smart phones [8]. It is suggested that this is a sustainable and reliable way of testing the workability of the device before assembly to avoid production losses. The method suggested has been proven effective and quick for the mass production scale of this device.

MEMS are, yet again, the centre of attention with the investigation of deep ion etching of Z-Cut Alpha Quartz [9]. Quartz is widely used in microelectromechanical systems (MEMS). MEMS quartz resonators especially are applied to sensors and serve as sensitive elements. Presented in this paper is a deep and high accuracy reactive ion etching method applied to a quartz resonator etching process with a Cr mask. In order to enhance the capability of deep etching and machining accuracy, three kinds of etching gas (C_4_F_8_/Ar, SF_6_/Ar and SF_6_/C4F_8_/Ar), bias power, inductively coupled plasma (ICP) power and chamber pressure were studied. Experimental results indicate that a Cr (chromium) mask can obtain a higher selectivity than an aluminium and titanium mask.

Concerning MEMS, it was shown that this approach can be used to design and manufacture micro-switches, which are suitable for small volume, low power consumption and high integration [10]. This paper reviews recent research of MEMS switches, pointing out the important performance indexes and systematically summarizing the classification according to driving principles. Then, a comparative study of current MEMS switches, stressing their strengths and drawbacks is presented, based on performance requirements such as driven voltage, power consumption, and reliability. 

Finally, the Special Issue concludes with the application of 3D printing into investment casting processes for achieving microstructure topography on the investment casted surfaces [11]. This is dependent on the accuracy and resolution of the 3D printed patterns and the ash and composition content after burning. This process has a great potential for production of small batches (customised products) with excellent surface finish due to the easily changeable 3D printed pattern.

As a conclusion, it is very important that the majority of the papers had specific targeted products and were developed to fulfil the design requirements of that specific product and application. This can provide more future directions and case studies for the precision engineering research community and the industry. In the field of micro/nano products, very often the design is limited to a specific manufacturing technology and/or even to a specific processing window. This is particularly true for MEMS products, and this explains the large number of MEMS devoted papers in the Special Issue. We would like in the future the opposite interaction between product and manufacturing technology, where the product based on its functional design requirements can dictate the process parameters and there is more than one available manufacturing process. The main challenge in employing such manufacturing systems is the complexity and interdisciplinary knowledge needed to develop and run such processes on an industrial scale. This additionally creates a demand to the engineering companies to employ high calibre specialists. Furthermore, material science also plays a vital role in the development of the micro/nano manufacturing processes and has become an inseparable part of these technologies.

It is amazing to see that the 11 papers included have all attempted to exploit the opportunity of producing micro and nano features, products, and systems, using different sorts of materials and different approaches. The presented papers reflect the state-of-the-art in this field and each one presents its own challenges. It is believed that the Special Issue has fulfilled the objectives to some extent, but there is a lot more to be shown and discussed. Therefore, it was decided that several Special Issues were to be created, devoted to specific technologies, applications, and equipment, and revealing the physics and chemistry behind the suggested technologies. We hope that the papers presented here will cast a light for future research and development. We also hope it will provide good reference and a handbook for industrial companies. The first 11 included papers are also expected to provide a drive for people interested and involved in micro/nano manufacturing technologies to gain the courage to attempt to publish their work with us and this way to continue their exploratory journey in the field.

This Special Issue has been made possible by joint efforts from the whole Micromachines Editorial Office. I would like to thank them for their kindly efforts, support, and patience throughout the process. 
